# The crosstalk between sonodynamic therapy and autophagy in cancer

**DOI:** 10.3389/fphar.2022.961725

**Published:** 2022-08-15

**Authors:** Yujie Zhang, Yuanru Zhao, Yuanyuan Zhang, Qingguang Liu, Mingzhen Zhang, Kangsheng Tu

**Affiliations:** ^1^ Department of Hepatobiliary Surgery, The First Affiliated Hospital of Xi’an Jiaotong University, Xi’an, Shaanxi, China; ^2^ School of Basic Medical Sciences, Xi’an Jiaotong University Health Science Center, Xi’an, Shaanxi, China

**Keywords:** sonodynamic therapy (SDT), sonosensitizers, autophagy, nanoparticles, cancer

## Abstract

As a noninvasive treatment approach for cancer and other diseases, sonodynamic therapy (SDT) has attracted extensive attention due to the deep penetration of ultrasound, good focusing, and selective irradiation sites. However, intrinsic limitations of traditional sonosensitizers hinder the widespread application of SDT. With the development of nanotechnology, nanoparticles as sonosensitizers or as a vehicle to deliver sonosensitizers have been designed and used to target tissues or tumor cells with high specificity and accuracy. Autophagy is a common metabolic alteration in both normal cells and tumor cells. When autophagy happens, a double-membrane autophagosome with sequestrated intracellular components is delivered and fused with lysosomes for degradation. Recycling these cell materials can promote survival under a variety of stress conditions. Numerous studies have revealed that both apoptosis and autophagy occur after SDT. This review summarizes recent progress in autophagy activation by SDT through multiple mechanisms in tumor therapies, drug resistance, and lipid catabolism. A promising tumor therapy, which combines SDT with autophagy inhibition using a nanoparticle delivering system, is presented and investigated.

## 1 Introduction

Ultrasound (US) has been widely used for both diagnostics and therapeutics in many fields, such as B-scan ultrasonography, bone repair, diabetic nephropathy, cancer therapy, immunotherapy, vaccination, and drug delivery (Cao et al., 2016; Escoffre et al., 2016; McHale et al., 2016; Padilla et al., 2016). Among these applications, the flexibility of US as an approach for noninvasively eradicating target solid tumors has recently drawn increasing attention (McHale et al., 2016). In cancer therapy, an ideal approach would be to apply a harmless stimulus that could lead to cytotoxic events at the target tumor tissue specifically and accurately. SDT, with its deep tissue penetration and high precision, has a great potential to become an ideal tumor therapy since sonochemical or sonophotochemical reactions, which lead to cytotoxicity under a controlled US irradiation with the help of certain chosen sonosensitizers (Liu et al., 2015). The major limitations of the sonosensitizers used in the SDT include poor delivery accuracy and high toxicity (Pelt et al., 2018). With the development of nanotechnology, nanoparticles (NPs)-based sonosensitizers were designed, which made use of NPs to deliver the sonosensitizers to target tissues or tumor cells with high specificity (Chen et al., 2014; Qian et al., 2016; Feng et al., 2019; Qu et al., 2020). The other problem encountered is that SDT can promote autophagy in tumor cells, which influences the efficiency of SDT. In other words, autophagy can assist tumor cell survival by recycling damaged organelles and misfolded proteins under different stress conditions (Helgason et al., 2013; White et al., 2015; Levy et al., 2017). Researchers have proposed to inhibit the SDT-induced autophagy in order to improve the efficiency of SDT improvement (Chen et al., 2014; Mahalingam et al., 2014; Vogl et al., 2014; Song et al., 2018; Feng et al., 2019; Qu et al., 2020).

In this review, the mechanism and sonosensitizers of SDT are firstly summarized. Next, the recent progress in tumor therapies with emphasis on SDT is introduced. The relationship between SDT and autophagy in tumor therapies and other metabolic pathways is also reviewed. To better present this study, an introduction to autophagy is also detailed. Finally, a promising tumor therapy, which combines SDT with autophagy inhibition using a nanoparticle delivering system, is presented and investigated.

## 2 Sonodynamic therapy

Before the use of US for therapeutic purposes, the application of light was considered an option for non-invasive therapy, which was referred to as photodynamic therapy (PDT). PDT is the predecessor of SDT. PDT involves three constituent elements: a light source, a photosensitizer, and the local tissue oxygen. Reactive oxygen species (ROS) are formed in the tumor cells and tissues with porphyrins upon irradiation with light (Rkein and Ozog, 2014). PDT can interfere with cytokine-mediated responses in tumor progression and metastasis and play a pivot role (Kaleta-Richter et al., 2019). However, since the light has only a limited penetration depth, PDT cannot be effectively applied to deep-seated tumors (Huang, 2005). At the same time, PDT highly relies on the ability of specific photosensitizing agents to concentrate in tumor cells (Kessel and Oleinick, 2009). Therefore, lacking a suitable photosensitizing agent with high efficiency, good sensitivity, wide applicability, and low toxicity severely constrain the research on PDT. In addition, the study showed that autophagy induced by PDT treatment could act as a mode of cell death after PDT (Buytaert et al., 2006; Kessel et al., 2006; Xue et al., 2007; Buytaert et al., 2008). However as a double-edged sword, autophagy can also promote tumor cell survival by compartmentalizing and recycling damaged tumor cell components after PDT, which significantly reduces the sensitivity and efficiency of PDT (Kessel et al., 2006; Kessel and Oleinick, 2009; Kaleta-Richter et al., 2019).

Based on PDT, SDT is gradually developed and optimized. The SDT is a new non-invasive tumor treatment method raised by Yumita et al. (1989) in 1989. When conducting research on PDT, they observed that some hematoporphyrin derivatives could also induce cell death under US irradiation (Yumita et al., 1989). It was then found that combined with US irradiation, some hematoporphyrin derivatives could act as sonosensitizers for tumor therapy, and this therapy was referred to as SDT. Research shows that most photosensitizers are sonosensitizers. Both PDT and SDT require the use of photosensitizer/sonosensitizers to trigger intracellular oxygen generation ROS to kill tumor cells. The SDT relies on the US and sonosensitizers present in the tumor tissue, with specific frequency and intensity, US irradiates the tumor site in deep tissue for a particular time, a lethal sono-damage such as necrosis and cell apoptosis by both mechanical stress and chemical reactions is caused and finally kills the tumor cells with high specificity and accuracy (Wang et al., 2013b; Parzych and Klionsky, 2014). Due to the deep penetration of US, SDT overcomes the major limitation of PDT. The SDT method is superior to the PDT method with deeper tissue penetration, higher precision, fewer side effects, and better patient compliance (Wang et al., 2013b; Pan et al., 2018b). The cytotoxic mechanisms of SDT in tumor treatment mainly include ultrasonic cavitation effect, singlet oxygen mechanism, mechanical damages, ultrasonic heating effect, apoptosis theory, and comprehensive effects of the mentioned mechanisms (Liu et al., 2015; Rengeng et al., 2017).

The major limitations of the sonosensitizers used in the SDT include poor delivery accuracy and high toxicity (Pelt et al., 2018). With the development of nanotechnology, nanoparticle-based sonosensitizers were designed, which make use of NPs to deliver the sonosensitizers to target tissues with high specificity (Chen et al., 2014; Qian et al., 2016; Feng et al., 2019; Qu et al., 2020). The other problem encountered is that SDT can promote autophagy in tumor cells, which inhibits the efficiency of SDT. It is because autophagy can assist tumor cell survival by recycling damaged organelles and misfolded proteins under different stress conditions (Helgason et al., 2013; White et al., 2015; Levy et al., 2017). Recently, the effect of autophagy induced by SDT in tumor treatment has drawn increasing attention (Wang et al., 2013b). Researchers have proposed to inhibit the SDT-induced autophagy in order to improve the efficiency of SDT (Chen et al., 2014; Mahalingam et al., 2014; Vogl et al., 2014; Song et al., 2018; Feng et al., 2019; Qu et al., 2020).

### 2.1 Mechanism of sonodynamic therapy

**FIGURE 1:**
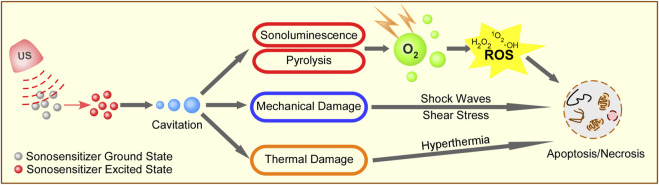
Schematic illustration of possible mechanisms of SDT. Ultrasound irradiation activates sonosensitizer from a ground state to an excited state to form cavitation around the surface of cancer cells. The energy from the collapse of cavitating bubbles can initiate sonoluminescent light, mechanical damage, and thermal damage to cancer cells. The energy can be transferred to the circumambient oxygen to produce a large amount of ROS, including singlet oxygen, peroxide, and hydroxyl radical, which subsequently mediate cell apoptosis and necrosis. At the same time, the cavitation also causes shock waves, shear stress, and hyperthermia to induce cell death.

The mechanisms of SDT include the generation of ROS, US mechanical damage, and thermal destruction. Figure 1 depicts the possible mechanisms of SDT.

#### 2.1.1 Generation of reactive oxygen species

ROS generation is the most widely accepted mechanism of SDT, and this theory focuses on the cancer cell apoptosis caused by ROS production induced by cavitation and sonosensitizers (Nora Frulio et al., 2010). Under the irradiation of US, sonosensitizers can be activated from the ground state to the excited state to generate ROS, including singlet oxygen and hydroxyl radicals. Acoustic cavitation plays an important role in activating sonosensitizers to generate ROS during the interaction of US with an aqueous environment. The cavitation process involves the nucleation, growth, and implosive collapse of gas-filled bubbles under certain US conditions. Cavitation can be classified into stable cavitation and inertial cavitation based on its nature. In stable cavitation, gas-filled bubbles oscillate with the surrounding liquid to create a mixing of the surrounding media. In inertial cavitation, the gas-filled bubbles grow to a near resonance size and expand before collapsing violently (Suslick, 1990). A considerable amount of energy is released in the implosion, leading to very high temperature (up to 10,000 K) and pressure (up to 81 MPa) in the microenvironment (Cavalli et al., 2018). The resultant extreme temperature and pressure may act as sonochemical reactors and lead to tumor cell necrosis (Misik and Riesz, 2000).

Two kinds of ROS can be generated by different reactions. The sonosensitizers at excited state can react directly with adjacent oxygen to turn hydrogen atoms to free radicals. Alternatively, the energy released by the sonosensitizers from the excited state to the ground state can be absorbed by the adjacent oxygen to produce high active singlet oxygens (^1^O_2_), which account for the primary SDT toxicity. In addition, sonoluminescence, a phenomenon referring to that light is generated when a solution is irradiated with US, is identified as another significant mechanism in generating ROS (Suslick et al., 1990; Yin et al., 2016). The sonoluminescent light can motivate the energy-matching photoactive sonosensitizers to a short-lived singlet state, which would lead to photochemical reactions (Didenko et al., 2000; Yin et al., 2016). Pyrolysis can be another possible mechanism for generating ROS (Danno et al., 2008). The extreme temperature and pressure during the implosion of gas-filled bubbles could generate free radicals in the following two ways: direct sonosensitizer breakdown or sonosensitizer reactions with H^+^ or OH^−^ produced by the thermolysis of water (Danno et al., 2008). Irreversible damage to targeted tumor cells, including cell membrane damage, DNA fragmentation, and mitochondrial membrane potential disturbance, can be induced by the high concentration of ROS (Kwon et al., 2019). Studies have shown that ROS activate autophagy through a variety of pathways to mediate cell survival or death. Especially, it was found that ROS induced by SDT triggered autophagy in a mitochondria-dependent minner (Qu et al., 2020).

#### 2.1.2 Ultrasound mechanical damages

The main factors causing US mechanical damage are cavitation effects and US radiation force (Yuan et al., 2015). It was reported that the mechanical pressure from US could lead to cell necrosis (Wang et al., 2003; Hiraoka et al., 2006). During the rapid collapsing of gas-filled bubbles under US irradiation, a considerable amount of energy is released to produce extreme temperature and pressure (Hiraoka et al., 2006). High shear stress and strong shock waves are generated, and they enhance the physical damage to cytomembranes, which ultimately lead to mechanical damage and tumor cell necrosis (Forbes et al., 2011).

#### 2.1.3 Thermal destruction

The increase of tissue temperature through absorption and transformation of the US mechanical energy can also lead to tumor cell necrosis (Xiong et al., 2020). For example, under the irradiation of appropriate US, the sonosensitizer mesoporous silica NPs can cause hyperthermia to kill tumor cells. Due to the high penetration and high energy concentration capabilities of US, SDT can cause thermal destruction to deep-seated tumors (Xiong et al., 2020).

### 2.2 Sonosensitizers with nanoparticle delivery system

In SDT, the sonosensitizers can be classified into two groups: organic and inorganic (Table 1). The organic sonosensitizers have high SDT efficiency but with poor pharmacokinetics, skin sensitivity, low stability, and poor SDT efficiency. The inorganic sonosensitizers have superior physical and chemical properties and can be multifunctional. However, the poor SDT efficiency and potential biosafety problems are the main challenges for inorganic sonosensitizers (Liang et al., 2020a).

**TABLE 1:** Summary of sonosensitizers and SDT conditions.

Sonosensitizer	Inorganic/organic	Biological model	US parameters	Biological effects	References
5-ALA	Organic	C6 glioma cells in rat	1.0 MHz, 10.0 W/cm^2^, 5 min	Deep seated intracranial glioma tumor size decreased; selective anti-tumour effect	Ohmura et al. (2011)
ATX-70	Organic	Mammary tumor cells (DMBA) in Sprague–Dawley rat	1.92 MHz, 1.0–5.0 W/cm^2^, 15 min	Significant tumor growth inhibition; strong synergistic effect (Ohmura et al., 2011)	Yumita et al. (2007)
DCPH-P-Na(I)	Organic	MKN-45 cells in mice	1.0 MHz, 1.0 W/cm^2^, 10 min	Potent sonotoxicity on tumor cells under US irradiation; significant tumor growth inhibition; potential for clinical treatment of cancers located deep in the human body without inducing skin sensitivity	Hachimine et al. (2007)
Porfimer sodium	Organic	Mammary tumor cells (DMBA) in Sprague–Dawley rat	1.92 MHz, 1.0–5.0 W/cm^2^, 15 min	Significant tumor growth inhibition; US intensity showed a relatively sharp threshold for the synergistic antitumor effect	Yumita et al. (2004)
Hematoporphyrin	Organic	Hepatoma-22 cells in mice	1.43 MHz, 2.0 W/cm^2^, 1 min	Strong cytotoxic effects; significant lipid peroxidation in tumor cells; tumor volume and weight were remarkably decreased; tumor cell ultra-structure was significantly damaged	Wang et al. (2011)
SF1	Organic	S180 sarcoma cells in mice	1.0 MHz, 1.2 W/cm^2^, 3 min	Tumor growth inhibition effect was enhanced with increasing US intensity; coagulated necrosis or metamorphic tissue with inflammatory reactions	Xiaohuai et al. (2008)
Hematoporphyrin derivatives	Organic	S180 sarcoma cells in mice	1.1 MHz, 1.5 W/cm^2^, 3 min	SDT induced morphologic changes; important factors inhibiting the tumor cell growth and even inducing tumor cell death: damage of cell structure, change of cytochrome C oxidase activity, degradation and missing of DNA	Liu et al. (2003)
Sinoporphyrin sodium	Organic	4T1 mouse xenograft model	1.0 MHz, 1.0 W/cm^2^, 2 min	Increased intracellular ROS production; change of membrane permeability; tumor growth and metastatic spreading inhibitions	Liu et al. (2016)
Protoporphyrin IX	Organic	Oral squamous carcinoma in mice	1.0 MHz, 0.89 W/cm^2^, 20% duty cycle, 15 min	Cell cycle arrested at G2/M phase; activate Fas-mediated membrane receptor pathway (regulated by p53) to induce apoptosis	Lv et al. (2017)
Chlorin e6 (Ce6)	Organic	Human breast cancer MDA-MB-231 cells *in vitro*	1.0 MHz, 0.5–2.0 W/cm^2^, 1 min	Inhibition on the proliferation of cancer cells; dose-dependently	Gao et al. (2010)
Rose bengal (RB)	Organic	Human colorectal adenocarcinoma cell line (HT-29 cells) in mice	1.0 MHz, 1.0 W/cm^2^, 2 min	Suitable for detecting tumor location and size; higher drug accumulation at the tumor site; enhanced ROS generation efficiency; enhanced SDT therapeutic efficacy with minimal side effects	Hou et al. (2020)
TiO_2_	Inorganic	C32 melanoma cells in mice	1.0 MHz, 1.0 W/cm^2^, 2 min	Tumor cell viability was significantly decreased; significant inhibition of tumor growth	Harada et al. (2011)
PMCS	Inorganic	Human breast cancer cells *in vitro*	1.0 MHz, 2.5 W/cm^2^, 50% duty cycle, 5 min	High sonosensitization efficacy and good stability; high ROS production and induced cellular destruction; high tumor inhibition efficiency	Pan et al. (2018a)
PtCu_3_	Inorganic	4T1 breast cancer cells in mice	35 kHz, 3.0 W/cm^2^, 10 min	Highly efficient ROS generation in SDT; significantly enhanced sonotoxicity to cancer cells; minimal toxicity to normal tissues at therapeutic doses	Zhong et al. (2020)
BaTiO_3_	Inorganic	*In vitro* cellular level evaluation and *in vivo* tumor xenograft assessment	1.0 MHz, 1.0 W/cm^2^, 50% duty cycle, 1 min	Built-in electric field catalyzed the generation of ROS; US triggered cytotoxicity promoted tumor eradication; high therapeutic biosafety.	Zhu et al. (2020a)
Porous silicon NPs	Inorganic	Lung Lewis carcinoma in mice	1.0 MHz, 1.0 W/cm^2^, 3 min	Inhibition on the growth of primary tumor site; slowed down the metastasis process.	Sviridov et al. (2017)
Copper-cysteamine	Organic	4T1 breast cancer cells in mice	1.0 MHz, 2.0 W/cm^2^, 3 min	Efficient production of ROS; significant inhibition of tumor growth; enhanced cavitation for tumor destruction	Wang et al. (2018)
Double-layer hollow manganese silicate (DHMS)	Inorganic	4T1 breast cancer cells in mice	1.0 MHz, 1.0 W/cm^2^, 50% duty cycle, 1 min	Highly effective ROS yield; production of oxygen in the tumor micro-environment to overcome the hypoxia of the solid tumor; good tumor inhibition ability and biosafety	Pan et al. (2020)

Currently, there are many limitations of sonosensitizers, which significantly influence the efficiency and sensitivity of SDT. The major challenges are the low drug delivery efficiency and limited generation of ROS (Hou et al., 2020). To make SDT practically applicable, thanks to the fast developing nanotechnology, nanoparticles-based sonosensitizers are introduced, which make use of nanoparticles to deliver the sonosensitizers to target tissues or tumor cells with high specificity and accuracy, and in the end, improve the outcomes of SDT (Pan et al., 2018b). Nano-sonosensitizers can be divided into two groups: nanoparticle as intrinsic sonosensitizer (TiO_2_, Ag/Pt, Si NPs, and polyhydroxy fullerene) and nanoparticle-assisted sonosensitizer (self-assembled, conjugated, embedded, or encapsulated) (Harada et al., 2011; Pan et al., 2018b; An et al., 2020a). Nano-sonosensitizers were summarized in Table 2 of Plenty of previous research has been conducted on NP-mediated SDT. As a typical semiconductor, TiO_2_ has drawn much attention, and TiO_2_ NPs are shown to have positive anti-tumor effects both *in vitro* and *in vivo* under US irradiation (Harada et al., 2011). Harada et al. (2011) reported that compared to 1 MHz of US irradiation alone, combined US irradiation of mice injected with TiO_2_ had a significant anti-tumor effect. However, the bio-distributions of traditional TiO_2_ NPs are hard to manipulate, and the acute and long-run toxicity is unsatisfactory (Chen et al., 2014). Therefore, searching for an efficient and sensitive sonosensitizer is urgently needed.

**TABLE 2:** Summary of nano-sonosensitizers.

NP	Drug carried/combination drug	Biological models	Target cells	Biological effects	References
TiO_2_	None	C32 melanoma solid tumors in mice	C32 melanoma cells	Tumor cell viability significantly decreased; remarkable antitumor effect	Harada et al. (2011)
Fe_3_O_4_ magnetic	Chitosan chloride (HTCC)/alginate	Gastric SGC7901/ADR tumor bearing mice	SGC7901/ADR gastric cancer cells	Excessive ROS accumulation and mitochondrial dysfunction; inhibition on the gastric tumor growth; reduced tumor volume	Li et al. (2016c)
Silver	Reduced graphene-oxide	Human cervical cancer (Hela CCL2) cell lines	Cervical cancer hela cells	Significant effects on the expressions of apoptotic and autophagy genes; accumulation of autophagosomes and autophagolysosomes; substantial generation of ROS	Yuan and Gurunathan (2017)
Gold	Protoporphyrin IX (PpIX)	Colon carcinoma tumor in male BALB/c mice	CT26 cancer cells	Higher tumor cell cellular uptake of PpIX; significant synergistic inhibitory effect on tumor growth; reduced tumor relative volume; increased average animal survival fraction	Shanei et al. (2012)
Porous silicon	Dextran (biopolymer)	Human lung cancer Hep2 cell lines	Hep2 cancer cells	Efficient uptake of the NPs by cancer cells; low cytotoxicity at high concentrations; the number of living cancer cells decreased	Sviridov et al. (2017)
Hollow polydopamine	Platinum NP, doxorubicin (DOX) and chlorine e6 (Ce6)	4T1 breast cancer cells in female Balb/C mice	4T1 cancer cells	Excellent biocompatibility with no toxicity to mice; effective delivery of drugs to target tumor cell mitochondria; relieved hypoxic state of the tumor site	An et al. (2020a)
Poly-methyl methacrylate	Meso-tetrakis (4-sulfonatophenyl) porphyrin (TPPS)	Human neuroblastoma SH-SY5Y cell lines	SH-SY5Y cells	Significant decrease in cancer cell proliferation; significant increase in necrotic and apoptotic cells; increased ROS production	Canaparo et al. (2013)
Poly (lactic-co-glycolic acid) (PLGA)	methylene blue (MB) and gadodiamide (Gd-DTPA-BMA)	MDA-MB-231 breast cancer cells in BALB/c nude mice	MDA-MB-231 cells	Combined therapeutic and diagnostic functionalities; better enrichment at the tumor site; promoted apoptosis triggered by US; good drug safety	Li et al. (2019)
Melanin	Folate and hematoporphyrin monomethyl ether	Human breast cancer MDA-MB-231 cells in female BALB/c nude mice	MDA-MB-231 cells	Enhanced photoacoustic imaging-guided SDT; accurate delivery of drugs to the tumor sites; engendered ROS-mediated cytotoxicity towards tumors	Huang et al. (2018)
Mesoporous silica	Doxorubicin (DOX) and Chlorin e6 (Ce6)	Breast cancer MDA-MB-2231 cells in female BALB/c nude mice	MDA-MB-2231 cells	High drug loading and delivery efficiency; targeted delivery and controllable activation potential; significant antitumor effect	Xu et al. (2020b)
Angiopep-2 peptide-modified liposomes	Ce6 and HCQ	GL261 glioma cells in female C57BL/6 mice	GL261 glioma cells	Selectively accumulated in the brain tumors during blood brain barrier opening; inhibited tumor growth and prolonged survival time	Qu et al. (2020)
Hollow mesoporous TiO_2_	HCQ and cancer cell membrane coating	Human breast cancer MCF-7 cells in nude mice	MCF-7 cells	Hide from macrophage phagocytosis; recognize and target the tumors by homologous targeting ability; elevated sensitivity of cancer cells to SDT	Feng et al. (2019)
Hollow mesoporous manganese trioxide (Mn_2_O_3_)	hyaluronic acid (HA) and HCQ	4T1 breast cancer cells in female BALB/c mice	4T1 cells	Significant lysosomal deacidification and autophagy blockade effects; selectively deliver HCQ to tumor sites; effective HCQ accumulation level at the target site	Zhang et al. (2019a)

Organic NP carriers for sonosensitizers are superior in high aqueous solubility, good bio-compatibility, satisfactory targeting specificity, and easy bio-degradation (Qian et al., 2016). Chen et al. (2017b) applied mitochondria targeting liposomes to carry hydrophobic sonosensitizer of hematoporphyrin monomethyl ether, which were released from the liposomes under US, and remarkably prevented the aggregation of sonosensitizers. Canaparo et al. (2013) reported that NPs delivered the sonosensitizers with high accuracy and enhanced the efficiency of carried sonosensitizers. Rui et al. (Hou et al., 2020) designed a sonosensitizer drug delivery system for rose bengal microbubbles. This drug delivery system was shown to have high drug loading content and good US imaging properties. Under US irradiation, the locations and sizes of the tumors were observed. More importantly, the rose bengal microbubbles were converted into rose bengal NPs through sonoporation effects *in situ* with US irradiation, which could bring many favorable effects, including high drug accumulation, and promoted generation efficiency of ROS. The high drug accumulation at the tumor site reduced the side effects, and the improved SDT efficiency showed great potential for cancer therapies.

The multifunctional NP systems with diagnostic and therapeutic functions have recently gained increasing attention in precision nanomedicine (Huang et al., 2018; Li et al., 2019). In the NP system investigated by Li et al. (2016a), the hydrophilic biodegradable polymeric NPs carried both sonosensitizer and a magnetic resonance contrast agent (Li et al., 2019). In order to target the tumor tissues with higher specificity and accuracy, the tumor-homing and penetrating peptide-F3 was used to decorate the surfaces of NPs. This designed F3 decorated poly lactic-co-glycolic acid NP showed a significantly higher tumor tissue concentration than non-targeted NP. More importantly, under satisfactory drug safety, the apoptosis caused by SDT with the designed multifunctional NP system was promoted. The results indicated a potential for further investigations and even clinical trials.

Huang et al (2018) carried out investigations on the combination of SDT with NP-assisted sonosensitizers under the guidance of photoacoustic imaging, and this novel therapy was expected to eradicate tumor cells accurately. The designed NP-assisted sonosensitizer had a core-shell structure and was based on poly lactic-co-glycolic acid. The core was constructed by integrating melanin NPs for photoacoustic imaging, and the hematoporphyrin monomethyl ether was used to build the shell to enhance photoacoustic imaging-guided SDT. Finally, tumor-targeting ligand folate was attached. The designed NP-assisted sonosensitizer had the following functions: high photoacoustic imaging contrast enhancement capability, accurate delivery of melanin NPs to target tumor tissues, and enhanced SDT performance. Both *in vitro* and *in vivo* experiments demonstrated the selective cytotoxicity effects of ROS induced by SDT on tumor cells with the assistance of NP-assisted sonosensitizer, which can promote the eradication of tumor tissues. Meanwhile, the toxicity of the designed NP-assisted sonosensitizer was evaluated, and this sonosensitizer was found to possess high biosafety.

Xu et al. (2020b) synthesized mesoporous silica NPs, and the NPs were loaded with doxorubicin and chlorin e6 as the sonosensitizer. Both *in vitro* and *in vivo* experiments were conducted to evaluate the anti-tumor effect of this NP-assisted sonosensitizer under SDT. The NPs were in the shapes of spheres with uniform sizes. The mesoporous structure led to high drug loading and delivery efficiency. On xenograft tumor-bearing mice, under US irradiation, the synthesized NP-assisted sonosensitizer showed a higher tumor suppression effect than doxorubicin combined with chlorin e6 or doxorubicin alone. The results suggested a potential for solid tumor therapy.

Zhang et al. (2019b) designed a mitochondria-targeted and US-activated NP delivery system for enhanced deep-penetration SDT. The built sonosensitizer IR780-based NPs showed effective surface-to-core diffusion *in vitro* and *in vivo*. With the guidance of US, the acoustic droplet vaporization effect significantly assisted the conveyance of IR780-NPs from the circulatory system to tumor tissues, and the acoustic wave force increased the penetration depth at the same time. Furthermore, the mitochondrial targeting capability of IR780-NPs improved the delivery accuracy. Following the mitochondrial targeting, the overproduction of ROS rendered cancer cells more susceptible to ROS-induced apoptosis. Meanwhile, IR780-NPs helped with photoacoustic and fluorescence imaging, which provided SDT guidance and monitoring potential.

### 2.3 Sonodynamic therapy combined with other therapy

Because of the advantages of SDT, it is an effective method for the treatment of a variety of diseases. Due to the complicated tumor microenvironment, many researchers have integrated SDT with other cancer treatment methods to achieve tumor eradication more effectively (Wan et al., 2016b). SDT can be applied as an adjunctive method to either chemotherapy, PDT, hyperthermotherapy, gas therapy, chemodynamic therapy, immunotherapy, or other therapies (Table 3) (Wan et al., 2016b; Liang et al., 2020a). Recent research suggested that favorable synergistic effects against tumor development and metastasis were achieved *in vitro* and *in vivo* when SDT was combined with other tumor therapies (Kondo and Kano, 1987; Gao et al., 2010; Wang et al., 2015b; Liang et al., 2020a).

**TABLE 3:** Category of combination therapy based on SDT in recent years.

Therapy	Materials	Sonosensitizers	*In vitro*	*In vivo*	US parameters	References
SDT/chemotherapy	Fe_3_O_4_–NaYF4@TiO_2_	TiO_2_	MCF-7	S180	1 W/cm^2^ at different time durations (0, 0.5, 1, 3, and 5 min)	Song Shen et al. (2014)
	Fe_3_O_4_@TiO_2_-doxorubicin	TiO_2_	MCF-7	S180	1 W/cm^2^; 3 min	Shen et al. (2015)
	MTN@DTX-CD	TiO_2_	MCF-7	S180	1 W/cm^2^; 40 s	Shi et al. (2015)
	HPDF nanomicelle	Hematoporphyrin	MCF-7, MCF-7/ADR	—	1 MHz, 1.5 W/cm^2^; 30 s	Wan et al. (2016a)
	HPDF nanoparticles	Hematoporphyrin	HepG2	HepG2	1.0 MHz, 1.5 W/cm^2^, 30 s	Liu et al. (2017)
	DOX@MSN-HA	MSN	MDA-MB-231	MDA-MB-231	30 s, 6 times of 5 s sonication, sonication interval = 1 min	Ding et al. (2017)
	O_2_MB-Gem	Rose Bengal	MIA PaCa-2, PANC-1	KPC mouse model	1 MHz, 30 s, 3 W/cm^2^, duty cycle = 50%, and PRF = 100 Hz	Nesbitt et al. (2018)
	DOX@HMONs-PpIX-RGD	PpIX	HCC	HCC	1.0 MHz, 50% duty cycle; 1 min	Li et al. (2018)
	DTX/X-NPs	Ce6	B16F10	B16F10	1.0 W/cm^2^ for 1, 3 and 5 min	Liu et al. (2018)
	HPCID (ICG@PCH@Dox@HA)	ICG	4T1	4T1	1.2 MHz for 60 s at 1, 2, and 3 W,	Wu et al. (2019)
	CDP@HP-T	Ce6	4T1	4T1	1.0 W/cm^2^; 3 min	An et al. (2020a)
	HPT–DOX	TiO_2_	4T1	4T1	1.0 MHz, 50% duty cycle, 0.5 W/cm^2^, 2 min	Liang et al. (2020b)
	O_2_MB-PTX-DOX and O_2_MB-PTX-RB	Rose Bengal	MCF-7	MCF-7	1 MHz, 30 s, 3 W/cm^2^, duty cycle = 50%, and PRF = 100 Hz	Logan et al. (2019)
	DOX@FeCPs	HMME	4T1, CT26	CT26	1.75 W/cm^2^ at different time durations (0, 0.5, 1, 3, and 5 min)	Xu et al. (2020a)
	MSN-DOX-Ce6	Ce6	MDA-MB-231	MDA-MB-231	0.5 W/cm^2^; 1 min	Xu et al. (2020b)
	CLH-5-ALA	5-ALA	4T1	4T1-luc cells	1 MHz, 3 W/cm^2^; 3 min	Xiao et al. (2021)
	GMCDS-FA@CMC (Au@mSiO_2_/Ce6/DOX/SLB-FA@CMC)	Ce6	3T3, C26	orthotopic colorectal tumors	—	Zhang et al. (2021c)
	CPDP (Ce6/PFP/DTX/PLGA)	Ce6	4T1	4T1	1–2 W/cm^2^ for different duration times	Zhang et al. (2021b)
	PCNP-DTX	Phycocyanin (PC)	MCF-7	S180	1 MHz, 0.75 W/cm; 2 min	Cao et al. (2021)
	DOX@PCN-224/Pt	TCPP	HUVEC, SKOV3 and CT26	CT26	1.0 MHz, 1.75 W/cm^−2^; 5 min	Ren et al. (2022)
	ZTC@M(ZIF-8@TPZ/Ce6@cytomembrane)	Ce6	AGS	AGS	1.0 MHz, 1.5 W/cm^2^; 3 min	Yu et al. (2022)
SDT/immunotherapy	MFC (membrane-coated Fe-PDAP/Ce6)	Ce6	4T1	4T1	1.0 MHz, 2 W/cm^2^, 50% duty cycle; 1 min	Jiang et al. (2022)
	PFCE@THPP_pf_-COPs	THPP	CT26	CT26	40 kHz, 2 W; 10 min	Yang et al. (2022)
	PEG-CDM-aPD-L1/Ce6	Ce6	B16–F10	B16–F10	2 MHz, 2.0 W/cm^2^, 20% duty cycle; 5 min	Huang et al. (2021)
	TiO_2_-Ce6-CpG	TiO_2_-Ce6-CpG	Hepa1-6	Hepa1-6	1.0 MHz, duty cycle: 50%, 1.0 W/cm^2^; 4 min	Lin et al. (2021)
	TIR@FITC-Nrf2-siRNA	IR780	CT26	CT26	1 MHz, 1.0 W/cm^2^, 50% duty cycle; 10 min	Wan et al. (2021)
SDT/PTT/Immunotherapy	CHINPs	HMME	4T1	4T1	1 MHz, 2.0 W/cm2, 50% duty cycle for different durations	Lin et al. (2022)
SDT-PDT	UCNPs@SiO2-RB	HMME	T24	—	2 W/cm^2^; 10 min	Xu et al. (2017)
	Fe@UCNP-HMME	HMME	T24	—	2 W/cm^2^; 5 min	Wang et al. (2019)
	UCNP@mSiO_2_(RB)-AgNPs	Rose Bengal (RB)	MRSA	—	2 W/cm^2^; 5 min	Zhao et al. (2020)
	TiO_2_	TiO_2_	PC3	—	—	Aksel et al. (2021)
	NSGQDs	Doped graphene quantum dots	MCF7	—	1 MHz, 1 W/cm^2^	Nene and Nyokong, (2021)
SDT/gas therapy	HMME/MCC-HA	HMME	MCF-7, NIH3T3	MCF-7	1 MHz, 1 W/cm^2^: 1 min	Feng et al. (2018)
	Lip-AIPH	Bubble liposomal systems	MCF-7	MCF-7	1.0 MHz, 2 min, 50% duty cycle	Lin et al. (2019)
	GCZ@M (GSNO/Ce6@ZIF-8@Mem)	Ce6	4T1	4T1	1 W/cm^2^; 3 min	An et al. (2020b)
	Au NR-mSiO_2_/AIPH	Au NR-mSiO_2_/AIPH	MCF-7	MCF-7	1.0 MHz, 0, 1.0, 1.5, 2.0, 2.5 W/cm^2^, duration (0, 3, 5, 7, 10 min)	Ye et al. (2021)
	OCN-PEG-(Ce6-Gd 3 +)/BNN6	Ce6	4T1	4T1	1 W/cm^2^, duty cycle = 50%, pulse frequency = 100 Hz, and frequency = 1 MHz; 5 min	Zheng et al. (2021b)
	Re-Cy/Re-CHO	Re_(I)_ tricarbonyl complexes	4T1	4T1	0.3 W/cm^2^, 3 MHz; 15 min	Zhu et al. (2022)
	T-mTNPs@L-Arg	TiO_2_	MCF-7	MCF-7	1 MHz, 1 W/cm^2^; 1 min	Zuo et al. (2022)
	BPPL (BP-Pt-PEI-L-Arg)	Black phosphorus	4T1	4T1v	1.0 MHz, 50% duty cycle, 1.5 W/cm^2^; 3 min	Cheng et al. (2022)
SDT/PTT	HMNCs (hematoporphyrin-melanin nanoconjugates)	Hematoporphyrin	4 T1	4 T1	US for 1, 3, 5, and 7 min	Zhang et al. (2021a)
	Cur-Au NPs-PEG	Cur-Au NPs-PEG	C540 (B16/F10)	C540 (B16/F10)	1.0 W/cm^2^; 1 min	Kayani et al. (2021)
	TiN (ultra-small titanium nitride) nanodots	TiN	4T1	4T1	40 kHz, 3.0 W/cm^2^	Wang et al. (2021b)
	CD@Ti3C2Tx HJs	Ti3C2Tx	4T1, MG-63, hMSCs	4T1	50 kHz, 3.0 W/cm^2^	Geng et al. (2021)
	H–Ti3C2-PEG NSs	Ti3C2 NSs (Ti3C2 MXene nanosheets)	4T1	4T1	<1 W/cm^1^; 10 min	Li et al. (2022)
SDT/CDT/chemotherapy	mZMD (mesoporous zeolitic-imidazolateframework@MnO_2_/doxorubicin)	mZM	HeLa	HeLa	1.0 MHz, 50% duty cycle 1.0 W/cm^2^; 1 min	Guan et al. (2022)
SDT/PDT/chemotherapy	RBNs (RB-loaded peptido-nanomicelles)	Rose Bengal (RB)	CNE-2Z	CNE-2Z	1.5 W/cm^2^, 3 min	Liu et al. (2019)
	OC (oleanolic acid-Ce6)	Ce6	PC9, 4T1	4T1	400 mW/cm^2^, 2 min	Zheng et al. (2021a)
SDT/PDT/PTT	PAIN (peptide amphiphile-ICG nanomicelles)	PAIN	MDA MB-231	MDA MB-231	1.5 W/cm^2^, 5 min	Liu et al. (2020)
SDT/Gas therapy/Immunotherapy	iCRET NPs	iCRET NPs	CT26, 4T1, and 4T1-Luc	CT26	5 min (100 s × 3 points)	Jeon et al. (2022)
SDT/Autophagy	CCM-HMTNPs/HCQ	HMTNPs	MCF-7	MCF-7	1 W cm^2^; 30 s	Feng et al. (2019)
	ACHL (angiopep-2 peptide-modified-liposomes co-loaded with Ce6 and HCQ)	Ce6	GL261	GL261	1.0 MHz, duty cycle: 20%, ultrasound power:1 W, burst interval time: 1 s, duration time: 60 s	Qu et al. (2020)
	PpIX/3-MA@Lip	PpIX	MCF-7	MCF-7	1.0 MHz, 1.5 W/cm^2^, 50% duty cycle	Zhou et al. (2021)

#### 2.3.1 Sonodynamic therapy with chemotherapy

As one of the commonly adopted clinical therapies against cancer, chemotherapy applies chemotherapeutic drugs with high toxicity to tumor cells. However, severe systemic side effects caused by chemotherapy have always been the major challenge for this tumor therapy. It is suggested that combining chemotherapy with SDT can lead to significantly improved synergistic therapeutic effects with reduced systemic toxicity (Shen et al., 2015; Shi et al., 2015; Nesbitt et al., 2018; Logan et al., 2019; Liang et al., 2020a; Xu et al., 2020b). Chemotherapy often leads to multidrug resistance, which can cause tumor recurrence and metastasis. Combining SDT with chemotherapy has shown great advantages in overcoming drug resistance owing to controlling the release of drugs and increasing cell membrane permeability (Wan et al., 2016a; Chen et al., 2017a; Liu et al., 2017; Li et al., 2018; Liang et al., 2020b; Guan et al., 2022). Moreover, SDT was found to promote cancer cells’ drug sensitivity by improving cellular internalization of chemotherapeutic drugs, activating the mitochondria-caspase signaling pathway, and down-regulating the expression of ATP-binding cassette transporters (Ding et al., 2017; Xu et al., 2020a; Ren et al., 2022). These functions enhanced the cytotoxicity of chemotherapeutic factors and therefore contributed to the promoted therapeutic efficiency (An et al., 2020a; Zhang et al., 2021b; Cao et al., 2021; Zhang et al., 2021c).

#### 2.3.2 Sonodynamic therapy with photodynamic therapy

SDT can also be combined with photodynamic therapy, named sono-photodynamic therapy (SPDT). SPDT could enhance intracellular ROS generation, severe mitochondria damage, cell migration inhibition, nuclear condensation, cell membrane permeability, and significantly trigger cell apoptosis (Wang et al., 2013a; Li et al., 2014a; Li et al., 2014b; Wang et al., 2015a; Liu et al., 2016; Atmaca et al., 2021). Nene and Nyokong (2021) designed a nanoplatform in which phthalocyanines (Pcs) were conjugated to nitrogen (NGQDs) and nitrogen-sulfur (NSGQDs) graphene quantum dots. The nanoparticles were irradiated with light for photodynamic therapy (PDT), ultrasound for sonodynamic therapy, and the combination of both in photo-sonodynamic therapy (PSDT). They found that only ^1^O_2_ was detected for PDT treatment. In contrast, both the ^1^O_2_ and ^•^OH radicals were evident after SDT and PSDT treatments, and the combination therapy showed improved ROS generation efficacy compared to the monotherapies (Nene and Nyokong, 2021). Aksel et al. (2021) found that malondialdehyde (MDA) levels were increased while superoxide dismutase activity (SOD), catalase (CAT), and glutathione (GSH) levels were decreased after TiO_2_-mediated SPDT (Aksel et al., 2021).

#### 2.3.3 Sonodynamic therapy with gas therapy

Gas therapy has attracted much attention as a novel “green” cancer treatment strategy in recent years (Chen et al., 2019). To date, several gases, such as nitrogen (N_2_), and nitric oxide (NO), carbon oxide (CO), carbon dioxide (CO_2_), have been involved in these therapeutic approaches (Chen et al., 2019). When SDT is combined with gas therapy for tumor treatment, the US triggers gas donors to release and produce highly toxic products or change the disease condition to promote the tumor suppression effects (An et al., 2020b; Cheng et al., 2022; Zhu et al., 2022; Zuo et al., 2022). The generated gas bubbles could be used as a powerful US contrast agent that greatly enhances the US contrast to guide cancer therapy. Some nanoplatforms, such as Lip-AIPH, Au NR-mSiO2/AIPH, OCN-PEG-(Ce6-Gd^3+^)/BNN6, and HMME/MCC-HA, have been explored, which integrated coordinated functions of diagnostics and therapy (Feng et al., 2018; Lin et al., 2019; Zheng et al., 2021b; Ye et al., 2021).

#### 2.3.4 Sonodynamic therapy with immunotherapy

As a promising therapeutic modality for cancer treatment, immunotherapy, which stimulates the host tumor-specific innate and acquires systemic immune responses to attack cancer cells, is different from other traditional cancer therapies that directly target malignant cells (Li et al., 2021; Liang et al., 2021). Furthermore, immunotherapy can ablate localized tumors, inhibit distant tumors, and suppress tumor metastasis. After immunotherapy, the host can form long-term immune memory to prevent tumor recurrence (Li et al., 2021; Liang et al., 2021). SDT can motivate immune responses by eliciting ICD (immunogenic cell death) induced by the production of cytotoxic ROSs to suppress tumor growth and prevent tumor recurrence effectively (Li et al., 2020; Cheng et al., 2021; Yin et al., 2021). PFCE@THPP_pf_-COPs designed by Yang et al. (2022) can attenuate tumor hypoxia and suppress tumor growth by inducing ICD of cancer cells. After combining with anti-CD47 immunotherapy, this synergistic treatment exhibited potent protective memory antitumor immunity to prevent tumor recurrence (Yang et al., 2022). Some smart SDT platforms that induced potent antitumor immune responses were designed in recent years, such as MON-PpIX-LA-CO_2_ (Yin et al., 2021), DYSP-C34 (Wang et al., 2021a), TiO_2_@CaP (Tan et al., 2021), membrane-coated Fe-PDAP/Ce6 (Jiang et al., 2022), Zn-TCPP/CpG (Zhu et al., 2020b), and so on. Immunotherapy has great potential to be combined with SDT to achieve more effective cancer treatments. Yue et al. (2019) combined noninvasive SDT with checkpoint blockade immunotherapy by the nanosonosensitizers HMME/R837@Lip to induce an anti-tumour response, and this combination arrested primary tumor progression and prevented lung metastasis. Other nanocarriers combining SDT with immunotherapy were also developed, such as TIR@FITC-Nrf2-siRNA (Wan et al., 2021), TiO_2_-Ce6-CpG (Lin et al., 2021), PEG-CDM-aPD-L1/Ce6 (Huang et al., 2021) and this combinatorial tumor therapeutics can robust anti-cancer immunity and long-term immune memory (Table 3).

#### 2.3.5 Sonodynamic therapy with other therapies

Materials with high absorbance in the NIR-II region could be applied in photo-induced cancer therapy. The photothermal effect could prolong blood circulation and improve the O_2_ supply, promoting ROS generation (Wang et al., 2021b; Geng et al., 2021; Li et al., 2022). The thermal effect in photothermal therapy can boost the efficiency of SDT and achieve synergistically enhanced therapeutic purposes (Zhang et al., 2021a; Kayani et al., 2021). At the same time, researchers have found that SDT can induce protective autophagy, which significantly reduces the efficiency of SDT, making it possible to combine SDT with autophagy to enhance the therapeutic efficacy (Feng et al., 2019; Liang et al., 2020a; Qu et al., 2020; Zhou et al., 2021).

## 3 Autophagy

### 3.1 Overview of autophagy pathway

**FIGURE 2:**
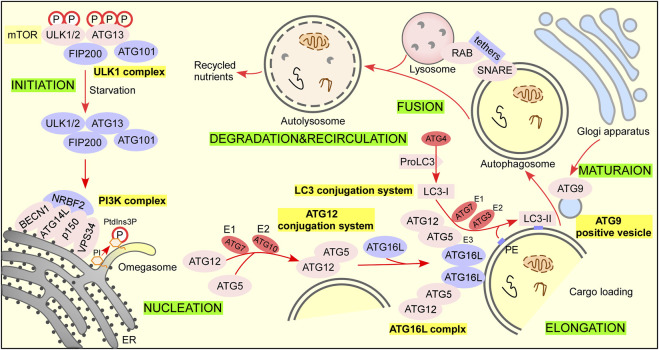
The autophagy pathway. There are 6 steps in the autophagy pathway. Step 1 Initiation: activation of ULK1 complex and multiple ATG proteins are engaged and localized to PAS. Under nutrient-rich conditions, mTORC1 phosphorylates ATG13 and ULK1/2 and blocks the interaction of ATG13 with ULK1/2, FIP200, and ATG101 to inactivate them (Hosokawa et al., 2009a; Hosokawa et al., 2009b; Park et al., 2016). When treated with rapamycin or under starvation conditions, mTORC1 dissociates from the complex and partially dephosphorylates these sites resulting in the complex anchors to a pre-autophagosomal structure (PAS) that recruits autophagy-related (ATG) proteins onto it to initial autophagy (Chan, 2009). Steps 2 Nucleation: ATG12 conjugation system and LC3 conjugation system are recruited to form phagophore. The ubiquitin-like protein ATG12 activated by the E1 enzyme ATG7 and E2 conjugating enzyme ATG 10 is irreversibly conjugated to ATG5 (Geng and Klionsky, 2008; Nakatogawa, 2013). The ATG12–ATG5 conjugate binds ATG16L1 with a lysine residue (K) in ATG5 to form the ATG16L1 complex (Hanada et al., 2007). The dimerization of ATG16L promotes membrane expansion (Ishibashi et al., 2011). ProLC3 is first cleaved by the cysteine protease ATG4 to expose their C termini glycine residue to form LC3-I (Satoo et al., 2009). LC3-I is activated by the E1 enzyme ATG7 and transferred to E2 enzyme ATG3, and the ATG16L complex exerts E3 enzyme activity that promotes the lipid conjugation of PE to LC3-I to form LC3-II for autophagosome formation (Kabeya et al., 2000; Fujita et al., 2008; Satoo et al., 2009; Nakatogawa, 2013). Step 3 Elongation: lipid enrichment supports a complex ubiquitin-like conjugation system that results in the conjugation of LC3 family members to the lipid phosphatidylethanolamine (PE) on phagophore. LC3 serves as a docking site for cargo adaptors that enable cargo loading into the AV. Step 4 Maturation: completion and transport of the autophagosome. ATG9-positive vesicles are delivered trans-Golgi apparatus, recycling endosome, and plasma membrane to contribute autophagosome maturation (Ravikumar et al., 2010; Takahashi et al., 2011; Orsi et al., 2012; Imai et al., 2016). Step 5 Fusion: autophagosome fuses lysosome to form autolysosome. Step 6 Degradation and recycling: degradation of cargo inside autolysosome and recycling of nutrients.

Autophagy can be divided into three types according to the role of lysosomes, including macroautophagy, microautophagy, and chaperone-mediated autophagy. Macroautophagy (referred to autophagy) is a process in which cells can form double-membraned autophagic vesicles named autophagosome that sequesters damaged organelles and misfold proteins and fuse with lysosome to form autolysosome for degradation (Figure 2) (Antonioli et al., 2017; Zhao et al., 2021). Microautophagy refers to a process in which misfolded proteins or damaged organelles are directly wrapped by lysosomal traps without forming autophagosomes. Molecular chaperone mediated autophagy refers to the process in which substrate protein is bound to lamp-2A receptor on lysosome by molecular chaperone such as HSP70, mediating its degradation (Bandyopachyay and Cuervo, 2008). Therefore, the autophagy process discussed in this paper is macroautophagy, referred to as autophagy.

#### 3.1.1 Initiation

Induction of autophagosome formation is regulated by the Unc-51-like kinase (ULK) complex, which is made up of a ULK family kinase (ULK1 and ULK2), autophagy-related gene 13 (ATG13), RB1-inducible coiled-coil 1 (RB1CC1/FIP200) and ATG101 (Hara et al., 2008; Chan, 2009; Zachari and Ganley, 2017).

#### 3.1.2 Nucleation, elongation, and maturation

The phosphatidylinositol 3-phosphate kinase (PI3K) complex consists of VPS34, BECN 1, ATG14L, and p150. Next, NRBF2 is recruited to the putative site of autophagosome formation (Matsunaga et al., 2009; Lu et al., 2014). Phosphatidylinositol (PI) in isolation membrane or omegasome is phosphorylated by the PI3K complex to produce phosphatidylinositol 3-phosphate (PtdIns3P), which recruits multiple PtdIns3P-binding proteins to regulate autophagosome formation. ATG12 conjugation system and LC3 conjugation system are involved in elongation. The ATG12-conjugation system includes ATG12, ATG7, ATG10, ATG5, and ATG16L. The LC3-conjugation system includes ProLC3, ATG4, LC3-I, ATG7, ATG3, and LC3-II (LC3-I/PE). The ATG9A/ATG2-WIPI1/2 trafficking system consisting of ATG9A, ATG2, and WIPI1/2 is also involved in autophagosome precursor formation.

#### 3.1.3 Fusion

The fusion of autophagosomes with functional endolysosomal compartments (early endosomes, late endosomes, and lysosomes) is required for autophagosome maturation, which is regulated by RABs, tethers (HOPS), and the SNARE complex (Itakura et al., 2012; Jiang et al., 2014; Tsuboyama et al., 2016; Shen et al., 2021).

#### 3.1.4 Degradation and recirculation of autophagosomal contents

When autophagosome fuses with lysosomes to form autolysosome, autophagic cargo such as misfolded protein and damaged organelles are degraded by lysosomal hydrolases as well as the inner membrane of the autophagosome is degraded into amino acids or peptides for recycling by cells (Mony et al., 2016; Yim and Mizushima, 2020).

### 3.2 Relationships between autophagy and tumor

Research has shown that the occurrence and development of tumors are closely related to autophagy. Autophagy is deemed an evolutionarily conserved catabolic process in mammalian cells (Glick et al., 2010; Amaravadi et al., 2019). In the autophagy process, a double-membrane autophagosome with a sequestrated intracellular component is delivered and fused with lysosomes for degradation (Parzych and Klionsky, 2014). Recycling these materials can provide energy for the survival of cells under a variety of stress conditions (Glick et al., 2010; White et al., 2015; Kocaturk et al., 2019). The protein aggregates and damaged organelles are also removed to ensure cell homeostasis and quality control (Glick et al., 2010; Kocaturk et al., 2019). Autophagy regulates various physiological functions such as stress resistance, cell death determination, and tissue remodeling. For human cancers, large-scale genomic analysis reveals that it is uncommon to lose or mutate core autophagy genes (Amaravadi et al., 2016). However, oncogenic events that activate autophagy and lysosome biogenesis are identified (Amaravadi et al., 2016). In addition, autophagy can promote cellular senescence and cell surface antigen presentation, which prevents genome instability and necrosis and finally prevents cancer (Glick et al., 2010). Autophagy impacts the interaction between the tumor and the host by assisting stress adaption and eliminating activation of adaptive immune responses. In addition, autophagy helps the crosstalk between the tumor and the stroma, which assists tumor growth under different stress conditions. Therefore, the factors influencing autophagy in cancer include microenvironment stress, starvation level, and the immune system (Amaravadi et al., 2016).

The dichotomous role of autophagy in tumor cells is elaborated in detail. On the one hand, in normal cells, the basal level of autophagy is crucial in ensuring protein quality control by removing misfolded proteins and preventing the accumulation of damaged DNA from maintaining genetic stability. As a result, the autophagy process can suppress the formation of tumor cells. At the early stage some tumors can undergo autophagic cell death (ACD) through the induction of autophagy by some anticancer drugs, in which progress autophagy plays a pro-death role (Amaravadi et al., 2016). On the other hand, autophagy can help tumor cells adapt to diverse adverse environments by providing nutrients and removing cytotoxic substances under stress, thus assisting tumor cell survival. In these circumstances, autophagy plays a pivotal cytoprotective role in promoting malignant tumors’ proliferation, invasion, and metastasis (White et al., 2015). In brief, autophagy acts as a double-edged sword (can have both pro-survival e and pro-death effects) in tumor occurrence, development, and metastasis (Helgason et al., 2013; Levy et al., 2017; Yun and Lee, 2018; Amaravadi et al., 2019).

## 4 The crosstalk of sonodynamic therapy and autophagy

### 4.1 Sonodynamic therapy induced autophagy

**FIGURE 3:**
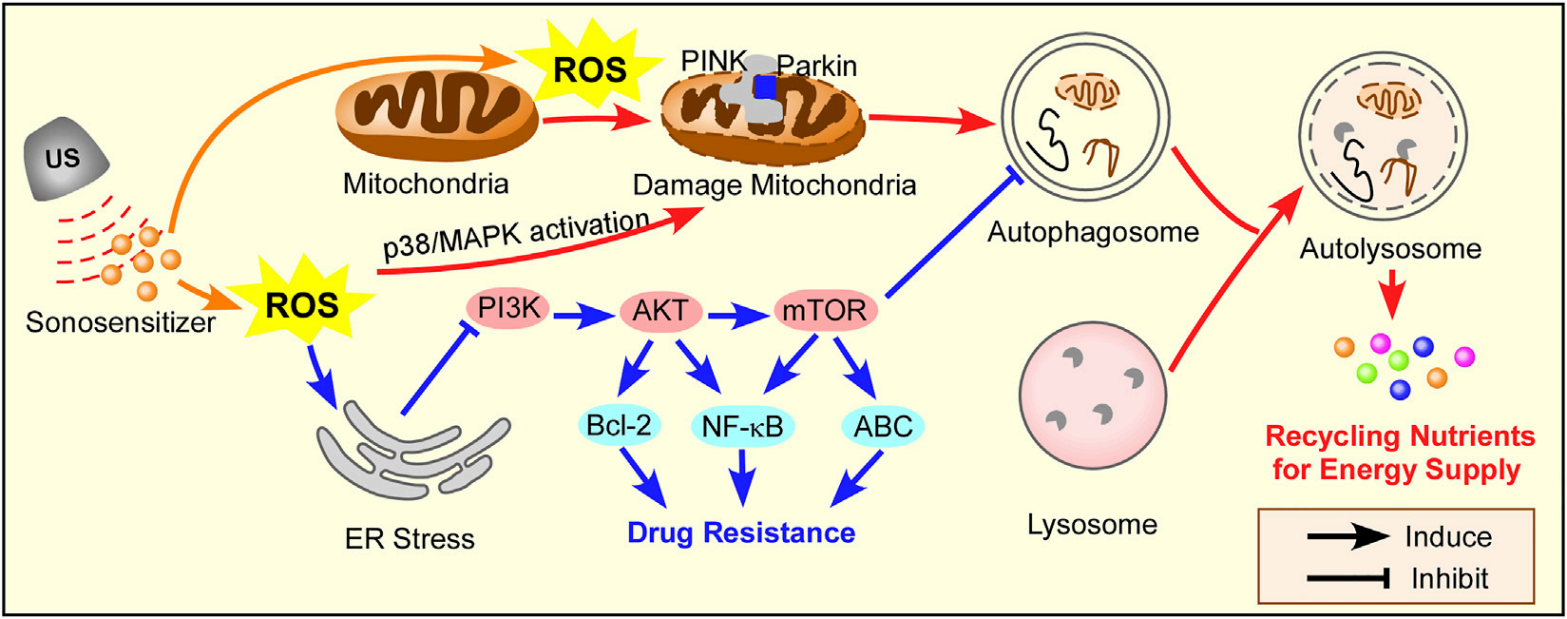
The mechanism of autophagy induction by SDT. ROS triggers mitochondria-apoptosis, which induces protective autophagy through the PINK/Parkin pathway in cancer therapy. SDT can inhibit chemotherapy sensitivity by ROS-induced ER stress, which activates autophagy in PI3K/AKT/mTOR pathway. Red arrow for cancer therapy, blue arrow for drug resistance in cancer therapy.

SDT triggers autophagy (or macroautophagy), which can be divided into two conditions in cancer therapy and lipid metabolism, as summarized in Figure 3. SDT-induced autophagy represents a double-edged sword, and a combination SDT with autophagy inhibition/activation strategies is summarized in Table 4.

**TABLE 4:** Summary the autophagy activation of SDT.

Type of cancer cell line	Sonosensitizer	Autophagy role	References
Murine leukemia L1210 cells	Protoporphyrin IX (PpIX)	Pro-survival	Wang et al. (2013b)
4T1 cells	Ce6	Pro-survival	Li et al. (2014a)
K562cells	Protoporphyrin IX (PpIX)	Pro-survival	Su et al. (2015)
THP-1-derived macrophage	Hypericin	Pro-death	Li et al. (2016b)
THP-1 macrophages	Berberin	Pro-death	Kou et al. (2017)
THP-1 macrophages	Hydroxysafflor yellow A	Pro-death	Jiang et al. (2017)
PTX-resistant PC-3 cells	—	Pro-survival	Wu et al. (2018)
MCF-7 cells	5-ALA	Pro-survival	Song et al. (2018)
MCF-7 cells	Hollow mesoporous titanium dioxide nanoparticles (HMTNPs)	Pro-survival	Feng et al. (2019)
GL261 cells	Ce6	Pro-survival	Qu et al. (2020)
4T1 cells	HMME@HMONs-3BP-PEG	Pro-death	Zou et al. (2021)
MCF-7 cells	Protoporphyrin IX (PpIX)	Pro-survival	Zhou et al. (2021)
B16-F0 mouse melanoma cells	Ce6	Pro-death	Zhang et al. (2022)

#### 4.1.1 In cancer therapy

The ultimate target of all anti-tumor therapies is to kill the tumor cells effectively and specifically. Cell death is classified into three types: apoptosis, autophagy, and necrosis (Kitanaka and Kuchino, 1999). Up to now, the major method in anti-tumor therapy is to induce apoptosis. However, tumor cells can escape from apoptosis with multiple pathways triggered by the anti-tumor treatments, among which autophagy is a novel cellular response and is attracting increasing attention (Hsin et al., 2011). Autophagy protects tumor cells from various stimuli, including amino acid deficiency, hypoxia, DNA and mitochondrial damage, and oxidative stress (Amaravadi et al., 2016). With SDT, the tumor cells suffer from various environmental stresses, and therefore autophagy is promoted to assist the tumor cell survival. As a result, the efficiency of SDT is significantly reduced. Many researchers have carried out investigations on SDT-induced autophagy in different tumor cells.

Su et al. (2015) investigated the interplay between apoptosis and autophagy induced by SDT in leukemia K562 cells. Under the protoporphyrin IX (PpIX)-mediated SDT, with the techniques of morphological observation and biochemical analysis, the mitochondrial-dependent apoptosis was noted. At the same time, SDT was observed to promote Autophagy in K562 cells and caused an increase in EGFP-LC3 puncta cells, conversion of LC3 II/I, formation of autophagosome, and co-localization between LC3 and LAMP2 (a lysosome marker). It was proposed that the SDT-induced autophagy is a cytoprotective mechanism because the autophagy inhibitor 3-MA or bafilomycin A1 was shown to suppress autophagy and enhance the SDT-induced apoptosis and necrosis. Experimental data suggested that the ROS caused by PpIX-SDT treatment may play an important role in inducing autophagy. Wang et al. (2013b) investigated the potential inductions of autophagy. Many signaling pathways are involved in the SDT-induced autophagy, such as those related to the control of mitochondria damage and ROS generation. Mitochondria can be a source of ROS and a target of oxidative damage during oxidation stress. In the experiments, the generation of ROS after SDT diffuses the whole cells, including mitochondria, and the accumulated ROS significantly affects the normal functions of the mitochondria. The damaged mitochondria co-localized rapidly with the autophagosome marker, which suggested that mitochondria damage can be one of the triggers for the induction of autophagy. In addition, ROS was also found to be involved in SDT-induced autophagy. Song et al. (2018) proposed the PINK1/Parkin-dependent signaling pathway to initiate mitophagy and autophagy in the human breast adenocarcinoma cell line MCF-7 cells subjected to SDT. The 5-aminolevulinic acid was used as the sonosensitizer. Excessive productions of ROS caused by SDT, together with the PINK1/Parkin-dependent signaling pathway, acted together to initiate mitophagy. The initiated mitophagy and autophagy helped protect the tumor cells against SDT-induced cell death. Qu et al. (2020) designed an “all-in-one” nanosensitizer platform that incorporated Ce6 and HCQ into angiopep-2 peptide-modified liposomes named ACHL. They found that SDT triggered mitophagy dependent on MAPK/p38 activation, which attenuated apoptosis in glioblastoma cells.

It is worth noting that SDT can enhance chemotherapy sensitivity and reverse drug resistance in tumor cells. At the same time, important clues has been emerged that autophagy promotes tumor drug resistance by involves the changes of apoptotic signals (Chang and Zou, 2020). The inhibition of Hedgehog (Hh) signaling pathway induces autophagy in BCR-ABL+ CML cells. Simultaneously inhibiting the Hh pathway and autophagy overcome CML drug resistance (Zeng et al., 2015). Many studies have shown that the addition of inhibitors of the PI3K/AKT/mTOR pathway can effectively enhance tumor therapy (Chang et al., 2013; Rangwala et al., 2014; Zeng et al., 2015). Wu et al. (2018) found that autophagy was induced in paclitaxel-resistant PC-3 cells after SDT treatment. A possible mechanism for promoting autophagy in paclitaxel-resistant PC-3 cells after SDT was the endoplasmic reticulum stress-mediated PI3K/AKT/mTOR signaling pathway. In the experiments, the observation of autophagy was realized by TEM and fluorescence microscopy. After SDT, the inhibition of PI3K/AKT/mTOR signaling pathway by endoplasmic reticulum stress-induced autophagy and autophagy reduced endoplasmic reticulum stress by eliminating the elimination of misfolded proteins and reactive oxygen species. It was further found that autophagy inhibition promoted endoplasmic reticulum stress, therefore down-regulating the PI3K/AKT/mTOR signaling pathway and finally leading to cell death (Wu et al., 2018).

### 4.2 Combining autophagy inhibition with sonodynamic therapy in cancer therapy

Considering the unfavorable effects of autophagy induced by SDT, many researchers suggested that the combination of SDT with autophagy inhibition can improve the efficiency of tumor therapy (Wang et al., 2010a). The role of autophagy in SDT-induced cytotoxicity in S180 cells was investigated by Wang et al., and an autophagy inhibitor study was performed. Through ample experiments, the autophagy inhibitors significantly promoted the SDT-induced cell death. Specifically, autophagy can participate in SDT-induced cell death, and the inhibition of autophagy at an early stage can promote the tumor treatment efficiency of SDT by the inducted apoptosis and necrosis. Autophagy was detected in the S180 cells treated with SDT under TEM. Double membrane-enclosed vacuoles containing damaged cellular components were observed with TEM. The autophagosomes and autolysosomes involved in this process were further confirmed by the immunofluorescence method. Western blot analysis showed that after SDT, autophagy flux happened in the early stage of cell damage. Kessel and Oleinick raised a similar point that within 1 hour following PDT, the PDT-induced autophagy can be detected. The results showed that with the application of autophagy inhibitors 3-methyladenine or Bafilomycin A1, after 1 hour following SDT, the loss of mitochondria membrane potential greatly increased, and therefore inhibiting autophagy can accelerate SDT induced cell death (Kessel and Oleinick, 2009).

Su et al. (2015) reported that intensified autophagy in human chronic myelogenous leukemia K562 cells was induced by protoporphyrin IX (PpIX) mediated SDT, and the induced autophagy was related to the up-regulation of Beclin-1 and the autophagic vacuoles observed in the SDT treated K562 cells. In the experiments, transfection of Beclin-1 shRNA inhibited the conversion of LC3 II/I and autophagic vacuoles significantly, which showed that autophagy was inhibited due to shRNA caused down-regulating of Beclin-1. In addition, compared with SDT only, the combined treatment with Beclin-1 shRNA led to more severe cytotoxicity. It was, therefore, demonstrated that SDT promoted autophagy of K562 cells significantly, autophagy might exert a self-protective effect against sonodamage for K562 cells, and finally, autophagy inhibition promoted cell apoptosis induced by SDT effectively.

The promising role of autophagy for drug development in cancer treatment was discussed. The state-of-art autophagy targeting methods and the potential role of autophagy in tumor immunity were thoroughly studied and reported. Autophagy suppressed tumor initiation but promoted advanced tumor growth. It was further found that autophagy inhibition could be sensitive and effective in advanced cancer therapy (Tanida, 2011). A variety of autophagy inhibitors in clinical trials and laboratory research were discussed, among which hydroxychloroquine (HCQ) treatment was found to be effective in cancer treatment (Shi et al., 2017). The combination of HCQ with other chemicals as the autophagy inhibitor for cancer therapy was also discussed (Goldberg et al., 2012; Cook et al., 2014; Mahalingam et al., 2014; Rangwala et al., 2014; Rosenfeld et al., 2014; Vogl et al., 2014). To conclude, the autophagy inhibitor plays a promising role in tumor treatment. Autophagy inhibitors were summarized in Table 5. Still, research on a suitable autophagy inhibitor with superior targeting, high efficiency, good sensitivity, and low toxicity is further needed. The initiation of autophagy by protoporphyrin IX (PpIX) mediated SDT in murine leukemia L1210 cells was examined by Wang et al. (2013b) Experiments showed that autophagy was protective for tumor cells after SDT and therefore impairment of autophagy can enhance the anti-tumor effect. Experimental data suggested that autophagy inhibition accelerated apoptosis and necrosis of SDT-treated tumor cells. A treatment combining autophagy inhibitors with SDT promotes tumor cell death and will be a promising method for cancer therapy.

**TABLE 5:** Summary of autophagy inhibitors.

Drugs	Target	Biological models	Status of the study	Biological effects	References
Chloroquine (CQ)	Lysosomal pH	Human breast cancer MCF-7 cells; human colorectal cancer cells	Approved by FDA; phase I clinical trial	Inhibition of protective autophagy by blocking autophagosome fusion and degradation; autophagy inhibition at the late stage of the pathway; evidence of preliminary antitumor activity	Sui et al. (2013)
Hydroxychloroquine (HCQ)	Lysosomal pH	Human esophageal, hepatocellular carcinoma, lung, and pancreatic cancer cells	Approved by FDA; phase I clinical trial	Inhibition of autophagosome fusion with lysosomes and autophagosome degradation; autophagy inhibition at the late stage of the pathway; induction of autophagic tumor cell death; safely dose escalated in cancer patients	Sui et al. (2013)
HCQ + tamoxifen	Lysosomes, estrogen receptor-ɑ (ERɑ)	Human breast cancer cells in female athymic mice	*In vivo* models; cancer cell lines; phase I clinical trial	Reduced drug resistance; *In vitro* and *in vivo* promotion of antiestrogenic therapy	Cook et al. (2014)
HCQ + temsirolimus	Lysosomes, mTOR pathway	Human renal carcinoma cell lines	Cancer cell lines	Induction of apoptosis and cell death; promotion of mitochondrial damage with mTOR down-regulation; tumor growth suppression	Lee et al. (2015)
3- methyladenine (3-MA)	Autophagosome formation, class III PI3K inhibitor	Human chronic myelogenous leukemia K562 cell line	Cancer cell lines	Inhibition on the formation of autophagosomes; autophagy inhibition at the early stage of the pathway; aggravated chromatin condensation; enhanced SDT-induced apoptosis and necrosis	Su et al. (2015)
Bafilomycin A1 (Ba A1)	Autophagolysosome formation, vacuolar-type H (+)-ATPase inhibitor	Murine sarcoma S180 cell line	Cancer cell lines	Inhibition on the fusion between autophagosomes and lysosomes; autophagy inhibition at the late stage of the pathway; enhanced SDT induced caspase-3 and PARP cleavage; enhanced SDT-induced cell death and anti-tumor effect	Wang et al. (2010a)
Monensin	Endocytic and lysosomal pH	Human non-small lung cancer NCI-H1299 cell line	Cancer cell lines	Inhibition on the fusion between autophagosomes and lysosomes; autophagy inhibition at the late stage of the pathway; enhanced cell cycle arrest and apoptosis; tumor growth suppression	Choi et al. (2013)
Wortmannin	Autophagosome formation, class III PI3K inhibitor	Hepatocytes from male wistar rats	Cells	Inhibition of autophagosome formation; potent inhibition of mammalian PtdIns 3-kinase; autophagy inhibition at the early stage of the pathway	Wang et al. (2010b)
2- (4-morpholinyl)-8-phenyl-chromone (LY294002)	Autophagosome formation, class III PI3K inhibitor	Chinese hamster ovary (CHO) cell line	CHO cell lines	Inhibition of autophagosome formation; promotion of rolipram-induced PDE4A4 aggregate/foci formation; potent inhibition of autophagic sequestration; autophagy inhibition at the early stage of the pathway	Christian et al. (2010)

Based on the above literature review, it is clearly identified that both SDT and autophagy inhibition has great potential in tumor therapy. To encounter the limitations of traditional sonosensitizers, the nanoparticles-based sonosensitizers, which make use of nanoparticles to deliver the sonosensitizers to target tissues or tumor cells, were introduced and investigated. The combination of SDT with nanoparticles-based sonosensitizers and autophagy inhibition proves to be a superior therapy with high specificity and accuracy and improves the outcomes of traditional tumor treatment methods. As an innovative tumor treatment concept, few researchers have carried out investigations in this field. However, as Qu et al. (2020) and Feng et al. (2019) suggested, this combinational tumor therapy method is sensitive, effective, and safe.

**FIGURE 4:**
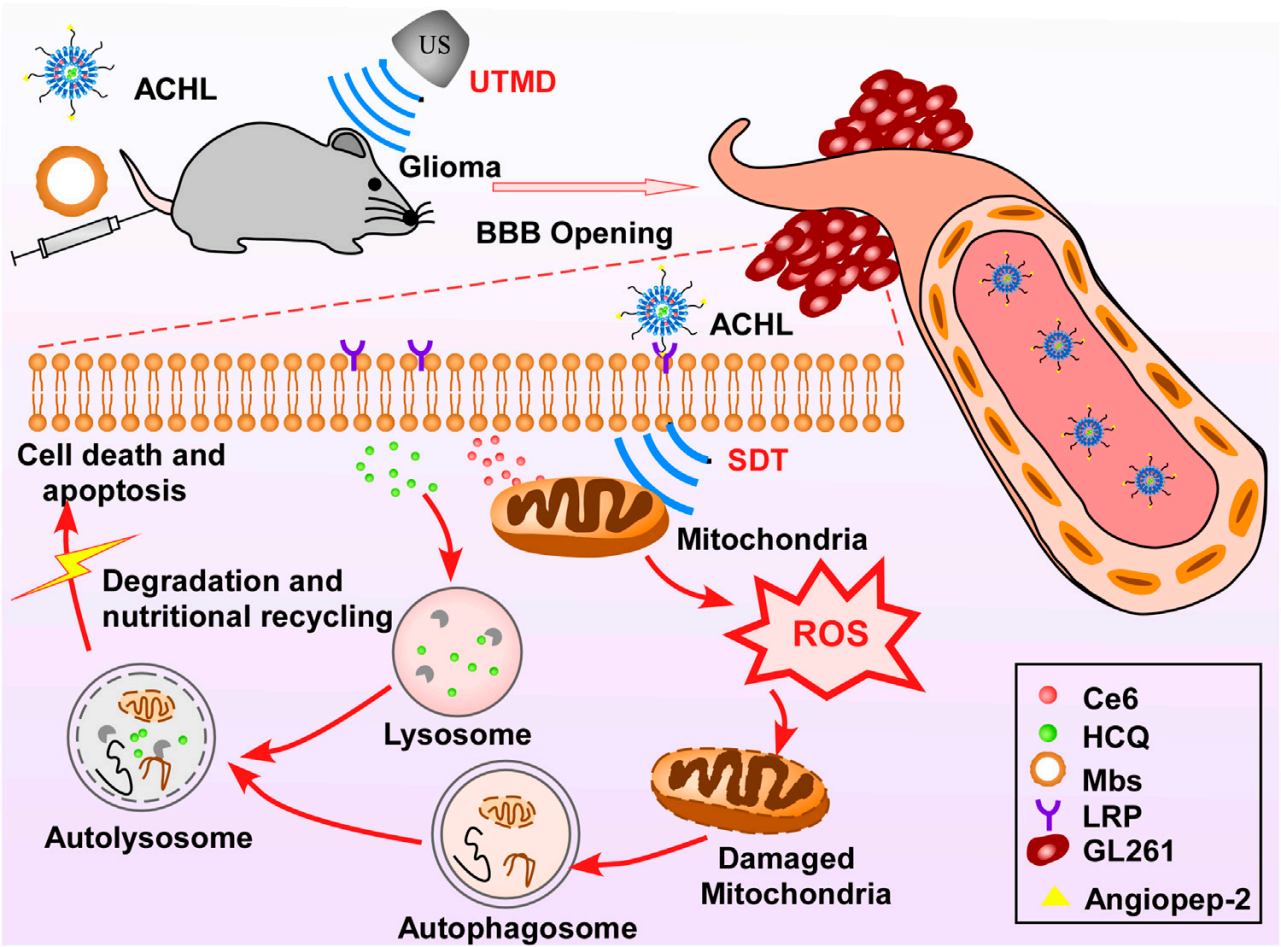
Schematic of “all-in-one” nanosensitizer platform. An “all-in-one” nanosensitizer platform by incorporating Ce6 and HCQ into angiopep-2 peptide-modified liposomes (designated ACHL) for orthotopic glioma theranostics was designed. An initial ultrasonic pulse (US1) destroyed the microbubbles and promoted the ACHL into the reversibly opened BBB, while a second ultrasonic stimulus (US2) generated the SDT effects. SDT-mediated mitophagy and its inhibition by HCQ were evaluated, along with the anti-glioma effects. The MAPK/p38 signaling pathway contributed to the progression of mitophagy induced by nanoCe6-SDT.

Qu et al. (2020) designed a novel “all-in-one” nanosensitizer and improved the SDT efficiency in glioma therapy. The “all-in-one” nanosensitizer incorporates the sonoactive chlorin e6 (Ce6) and hydroxychloroquine (HCQ) into angiopep-2 peptide-modified liposomes (APL), in which APL acts as the NP platform for drug delivery, Ce6 acts as the sonosensitizer, and HCQ acts as the autophagy inhibitor. APL can selectively accumulate in the glioma cells in the brain, which is a good strategy for precise drug delivery and can help minimize the side effects of the applied drugs on orthotopic cells. As the autophagy helps tumor cells adapt to oxidative injury and other stress after SDT by recycling the damaged mitochondria, the autophagy inhibitor HCQ prevents the above process and significantly enhances the SDT therapeutic effect. The mechanism of autophagy inhibitor improving SDT efficiency was proposed in this review as the autophagy blockage can enhance oxidative damage and increase the apoptosis response (Figure 4).

**FIGURE 5:**
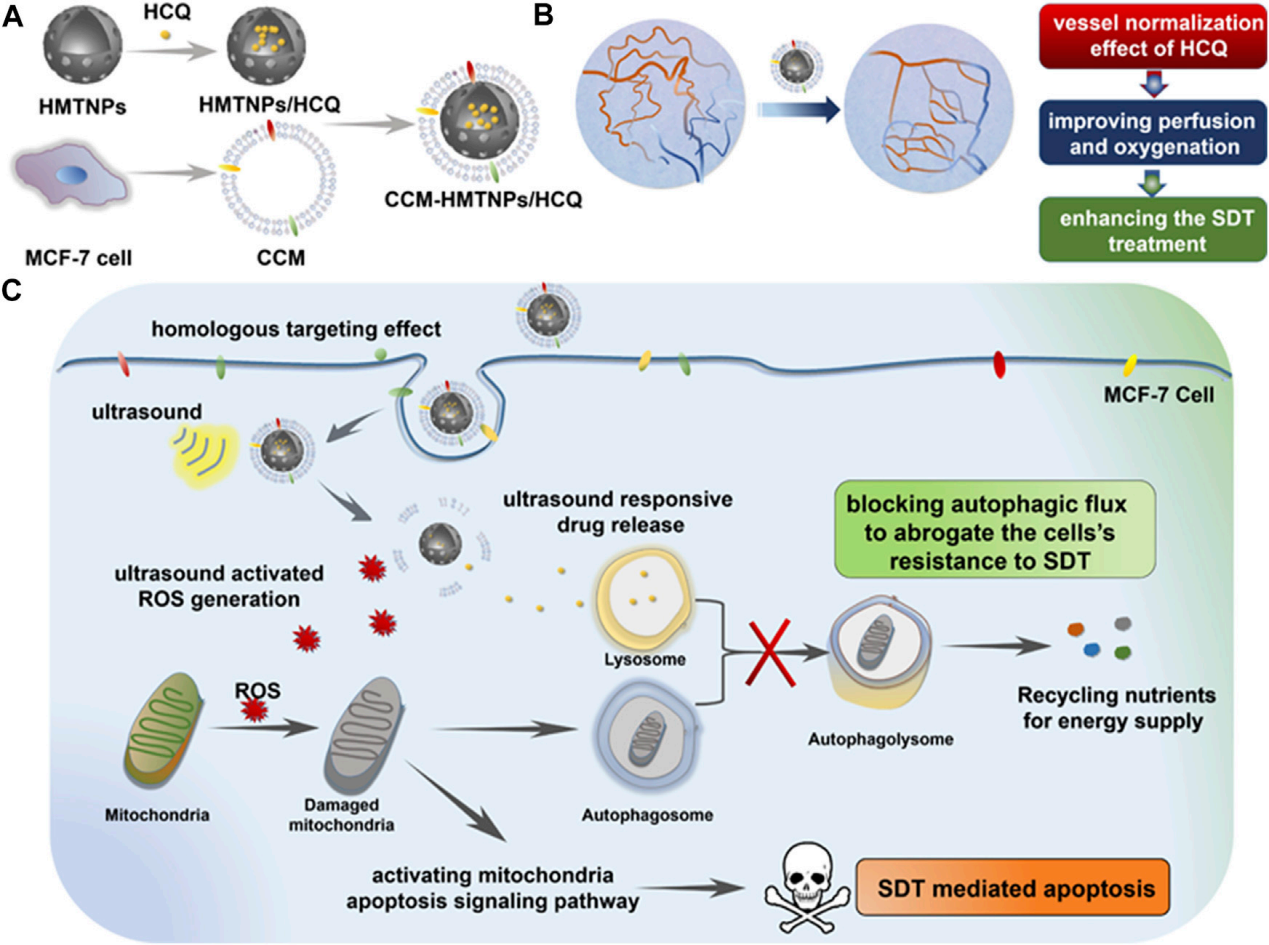
Schematic of the Cancer Cell Membrane Biomimetic Nanoplatform. **(A)** formulation of CCM-HMTNPs/HCQ, **(B)** vessel normalization effect of HCQ for enhancing the oxygen-dependent SDT treatment, and **(C)** schematic mechanism of CCM-HMTNPs/HCQ for enhanced SDT on breast cancer *via* autophagy regulation strategy. Copyright 2019, ACS Applied Materials & Interfaces.

A similar therapeutic method was reported by Feng et al. (2019), in which the sonosensitizer hollow mesoporous titanium dioxide NPs (HMTNPs), along with the autophagy inhibitor HCQ, were combined and coated with cancer cell membranes (CCM). The biomimetic CCM coating allowed the CCM-HMTNPs/HCQ drugs to be delivered to target tumor cells. Under US irradiation during SDT, HCQ was released to block the autophagy process. Moreover, the vessel normalization effect of HCQ improved the tumor cell hypoxia situation, which significantly promoted the oxygen-dependent HMTNPs-mediated SDT (Figure 5). It was concluded that the CCM-HMTNPs/HCQ drugs highly enhanced the sensitivity and efficiency of SDT on breast tumor cells (Feng et al., 2019).

**FIGURE 6:**
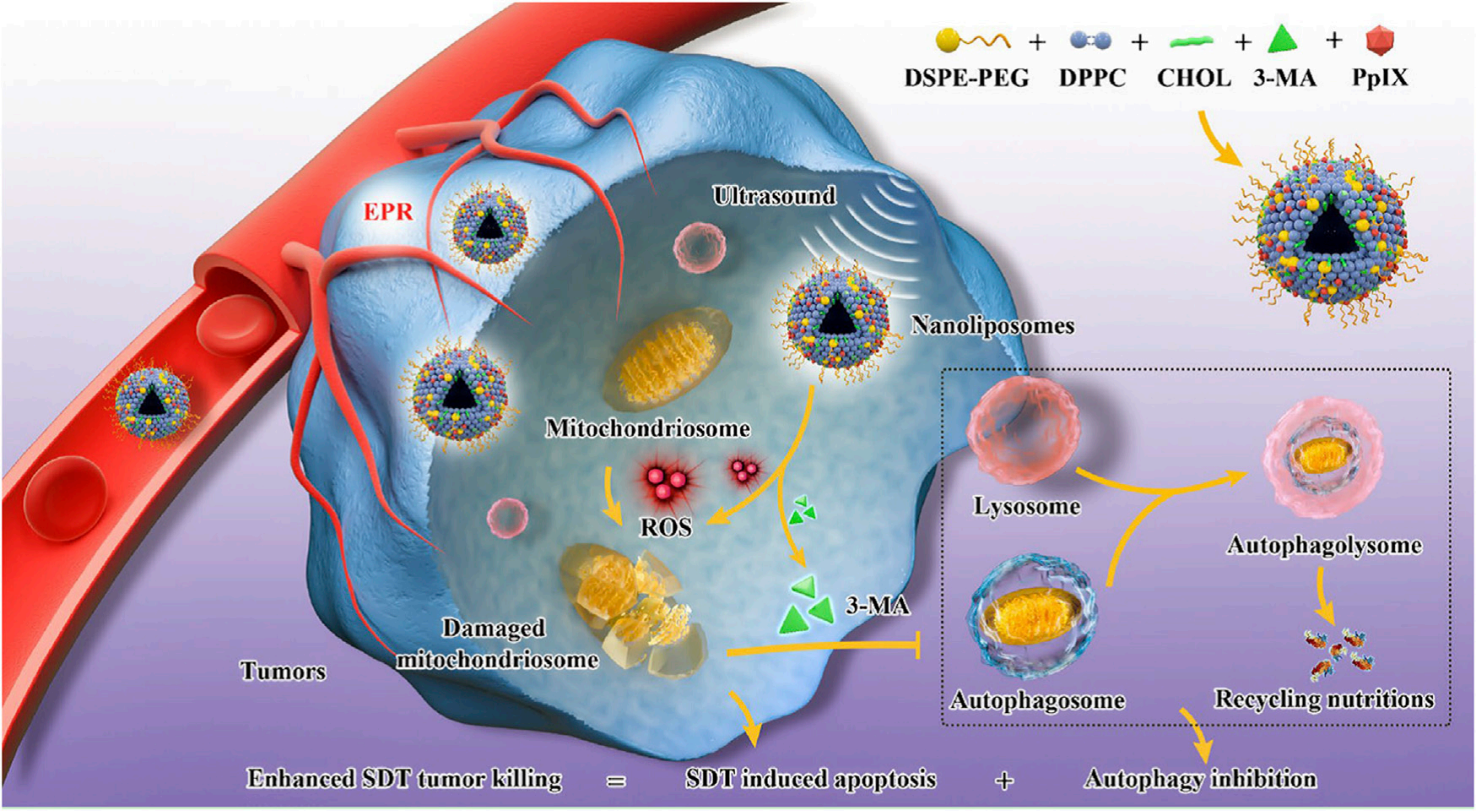
Engineering PpIX/3-MA@Lip nanosonosentizer for synergistic SDT nanotherapeutics and autophagy blockage on combating cancer. The synthetic procedure of PpIX/3-MA@Lip nanosonosensitizers and schematic illustration of “all-in-one” strategy for cellular mechanism on SDT-induced cytoprotective autophagy and autophagy inhibition-enhanced antitumor efficacy of SDT. Enhanced production of intracellular ROS radicals by PpIX sonosensitizers-based SDT induced cytoprotective pro-survival autophagy. The integrated 3-MA inhibited the formation of autophagosomes in early-phase autophagy to eliminate the recycling nutrients for fulfilling the needs of cancer-cell adaptation and growth, which significantly induced the cancer-cell apoptosis and death. Copyright 2021, Journal of Nanobiotechnology.

Zhou et al. (2021) constructed an “all-in-one” nanoliposomes co-encapsulating sonosensitizers protoporphyrin IX (PpIX) and autophagy inhibitor 3-MA. It has been demonstrated that SDT induced cytoprotective pro-survival autophagy with alleviating apoptosis through the MAPK signaling pathway. Combining SDT and autophagy blockage significantly decreased the cell resistance to intracellular oxidative stress and resulted in a remarkable synergistic effect on cancer therapy (Figure 6).

Therefore, applying autophagy inhibitors to improve the sensitivity of SDT in tumor therapy is expected to provide a new and promising strategy to increase the SDT efficiency and sensitivity.

## 5 Summary and outlook

Originated and developed from PDT, SDT is a novel non-invasive tumor therapy. The advantages of SDT include deep tissue penetration, high precision, low side effects, and good patient compliance. SDT refers to the “cavity effect” generated by the irradiation of ultrasound at a specific frequency on tumors in deep tissues. Under US irradiation, the sonosensitive accumulated by tumor cells changes them from the ground state to the excited state and reacts with surrounding oxygen molecules to produce a large number of ROS, resulting in tumor cell death. SDT has made great progress in the treatment of tumors, and there have been many studies on the treatment of pancreatic cancer, liver cancer, breast cancer and other deep tumors. How to improve the efficiency of SDT is still the focus and difficulty of sonodynamic research.

Firstly, the efficiency of SDT highly relies on the sonosensitizer, and the major challenge for the application of SDT lies in the poor sonosensitizer delivery accuracy. With the development of nanotechnology, NPs are introduced to deliver the sonosensitizers to target tissues or tumor cells with high specificity. Many researchers have investigated the suitable NPs to be used as target delivery carriers, which are reviewed in this paper. Secondly, the main mechanism of SDT is that US uses oxygen in tumor cells to produce a large number of ROS to destroy tumor cells. Increasing the production of ROS can fundamentally improve the efficacy of SDT. Studies have shown that reactive oxygen species can activate autophagy through a variety of pathways, degrade damaged proteins and organelles in cells, and mediate cell survival or death. Wang et al. (Wang et al., 2013a; Wang et al., 2013b) found that SDT activated autophagy while inducing apoptosis, and the activated autophagy inhibited apoptosis and protected tumor cells in 2013. Many successive studies have found that SDT-activated autophagy depends on ROS production, and activated autophagy helps tumor cells survive, which was summarized in this paper. It is speculated that avoiding the activation of protective autophagy during SDT can effectively improve the treatment sensitivity. State of the art therapeutic technique is the combination of SDT with nanoparticles-based sonosensitizers together with autophagy inhibition, which proves to be a superior therapy with high specificity and accuracy and improves the outcomes of traditional tumor treatment methods. Few investigations have been done in this area and are summarized in this review. Qu et al. (2020) used Angiopep-2 peptide modified liposomes to deliver Ce6, an acoustic sensitizer, and HCQ, an autophagy inhibitor, simultaneously to improve the acoustic dynamic therapy effect of glioma. Feng et al. (2019) used hollow mesoporous titanium dioxide nanoparticles to deliver the autophagy inhibitor HCQ and improve the SDT effect of breast cancer. Zhou et al. (2021) used liposomes to deliver acoustic sensitizer PpIX/autophagy inhibitor 3-MA synchronously, improving the therapeutic effect of acoustic dynamics on breast cancer.

Increasing the accumulation of sonosensitive agents in tumor sites using nanocarriers can increase the production of ROS, and then fundamentally improve the efficiency of sonodynamic therapy. The increase of ROS activated protective autophagy to inhibit the SDT efficacy. Therefore, it is speculated that the combination of targeted delivery of sonosensitizer and inhibition of autophagy can not only effectively kill tumor cells by increasing the production of ROS, but also avoid the activation of protective-autophagy by ROS. The findings in this review suggest that this is a promising tumor therapy, and more investigations need to be carried out in this area.
